# Prospective comparative multi-centre study on imported *Plasmodium ovale wallikeri* and *Plasmodium ovale curtisi* infections

**DOI:** 10.1186/s12936-018-2544-6

**Published:** 2018-10-30

**Authors:** Gerardo Rojo-Marcos, José Miguel Rubio-Muñoz, Andrea Angheben, Stephane Jaureguiberry, Silvia García-Bujalance, Lina Rachele Tomasoni, Natalia Rodríguez-Valero, José Manuel Ruiz-Giardín, Joaquín Salas-Coronas, Juan Cuadros-González, Magdalena García-Rodríguez, Israel Molina-Romero, Rogelio López-Vélez, Federico Gobbi, María Calderón-Moreno, Esteban Martin-Echevarría, Matilde Elía-López, José Llovo-Taboada, TropNet Plasmodium ovale investigator group, Mar Lago-Nuñez, Fancesco Castelli, Jerónimo Jaquetti, Carles Alonso, Daniel Camprubí, Miguel Górgolas-Hernández-Mora, Guillermo Cuevas-Tascón, Ana Pérez-Ayala, María Teresa Guzmán-GarcíaMonge, Mónica Ribell-Bachs, Nuria Serre-Delcor, Yasmina Martín-Martín, Germán Ramírez-Olivencia, Rosario Cogollos-Agruña, Francisco Jesús Merino-Fernández, Antonia García-Castro, Purificación Cantudo-Muñoz, Moncef Belhassen-García, Pablo Martín-Rabadán, Purificación Rubio-Cuevas, Elena Trigo-Esteban, Juan Arévalo-Serrano

**Affiliations:** 1 Hospital Universitario Príncipe de Asturias, Ctra de Meco s/n, 28805 Alcalá de Henares, Madrid Spain; 2 Instituto de Salud Carlos III, Majadahonda, Madrid Spain; 3 Ospedale Sacro Cuore - Don Calabria, Negrar, Verona Italy; 4 Hôpital Pitié-Salpêtrière, Assistance Publique des Hôpitaux de Paris (APHP), Paris, France; 5 Hospital Universitario La Paz-Carlos III, Madrid, Spain; 6 Spedali Civili Hospital, University of Brescia, Brescia, Italy; 7 Hospital Clinic, Barcelona, Spain; 8 Hospital Universitario de Fuenlabrada, Madrid, Spain; 9 Hospital del Poniente-El Ejido, Almería, Spain; 10 Hospital General Universitario de Valencia, Valencia, Spain; 11 Hospital Vall D´Hebron-Drassanes, Barcelona, Spain; 12 Hospital Universitario Ramón y Cajal, Madrid, Spain; 13 Hospital Universitario Gregorio Marañón, Madrid, Spain; 14 Hospital Universitario de Guadalajara, Guadalajara, Spain; 15 Complejo Hospitalario de Navarra, Pamplona, Navarra Spain; 16 Complejo Hospitalario Universitario de Santiago de Compostela, A Coruña, Spain

**Keywords:** *Plasmodium ovale curtisi*, *Plasmodium ovale wallikeri*, Comparative study, Thrombocytopenia, INR, Antimalarials, Diabetes mellitus

## Abstract

**Background:**

Few previous retrospective studies suggest that *Plasmodium ovale wallikeri* seems to have a longer latency period and produces deeper thrombocytopaenia than *Plasmodium ovale curtisi*. Prospective studies were warranted to better assess interspecies differences.

**Methods:**

Patients with imported *P. ovale* spp. infection diagnosed by thick or thin film, rapid diagnostic test (RDT) or polymerase chain reaction (PCR) were recruited between March 2014 and May 2017. All were confirmed by DNA isolation and classified as *P. o. curtisi* or *P. o. wallikeri* using partial sequencing of the ssrRNA gene. Epidemiological, analytical and clinical differences were analysed by statistical methods.

**Results:**

A total of 79 samples (35 *P. o. curtisi* and 44 *P. o. wallikeri*) were correctly genotyped. Males predominate in wallikeri group (72.7%), whereas were 48.6% in curtisi group. Conversely, 74.3% of curtisi group were from patients of African ethnicity, whilst 52.3% of Caucasians were infected by *P. o. wallikeri*. After performing a multivariate analysis, more thrombocytopaenic patients (p = 0.022), a lower number of platelets (p = 0.015), a higher INR value (p = 0.041), and shorter latency in Caucasians (p = 0.034) were significantly seen in *P. o. wallikeri*. RDT sensitivity was 26.1% in *P. o. curtisi* and 42.4% in *P. o. wallikeri*. Nearly 20% of both species were diagnosed only by PCR. Total bilirubin over 3 mg/dL was found in three wallikeri cases. Two patients with curtisi infection had haemoglobin under 7 g/dL, one of them also with icterus. A wallikeri patient suffered from haemophagocytosis. Chemoprophylaxis failed in 14.8% and 35% of curtisi and wallikeri patients, respectively. All treated patients with various anti-malarials which included artesunate recovered. Diabetes mellitus was described in 5 patients (6.32%), 4 patients of wallikeri group and 1 curtisi.

**Conclusions:**

Imported *P. o. wallikeri* infection may be more frequent in males and Caucasians. Malaria caused by *P. o. wallikeri* produces more thrombocytopaenia, a higher INR and shorter latency in Caucasians and suggests a more pathogenic species. Severe cases can be seen in both species. Chemoprophylaxis seems less effective in *P. ovale* spp. infection than in *P. falciparum,* but any anti-malarial drug is effective as initial treatment. Diabetes mellitus could be a risk factor for *P. ovale* spp. infection.

## Background

Malaria remains a global health problem with more than 212 million new cases in 2016 and 429,000 annual deaths worldwide, mostly driven by *Plasmodium falciparum* and *Plasmodium vivax*, to which most of the research resources are devoted [1].

Conversely, *Plasmodium ovale* spp. infection can be considered a neglected disease and little is known on its real incidence, geographic distribution, global disease burden or interactions with other *Plasmodium* species. The main problems for its study have been that it is frequently misdiagnosed because of usual submicroscopic or low parasitaemia levels, low performance of malaria rapid diagnostic tests (RDT) [2] and a majority of mixed infections with other *Plasmodium* spp. [3].

Nowadays, polymerase chain reaction (PCR) techniques have expanded the knowledge on *P. ovale* spp. epidemiology reporting infections in most of sub-Saharan Africa, Southeast Asia, and the Indian subcontinent [4–6], but not in the Americas, with prevalences as high as 15% in zones of Nigeria or Papua New Guinea [3]. In addition, severe complications, such as acute respiratory distress syndrome (ADRS) [7], spleen rupture, severe anaemia, or death may occur in patients with *P. ovale* spp. malaria [8]. Lastly, the incidence of imported *P. ovale* spp. infection seems to be increasing among travellers returning from West Africa [9–11], in whom chemoprophylaxis failed to work more frequently [12] compared to *P. falciparum* or *P. vivax* [13].

Since the study by Sutherland et al. was published in 2010, *P. ovale* is considered to comprise two genetically different subspecies, named *P. ovale curtisi* and *P. ovale wallikeri* that could have diverged 1–2 million years ago in their evolution [4]. They both are morphologically identical and cannot be differentiated by microscopy. Increasing information from different studies in endemic countries support that both subspecies co-circulate in Africa and Asia and are unable to recombine genetically [14–17].

Once established the genetic differences, the next question was to find out if there were any other differences in epidemiology, microbiology or clinical features between both subspecies. *Plasmodium o. curtisi* and *P. o. wallikeri* do not seem to differ in parasitaemia levels [5, 9, 13] and the absence of Schüffner’s stippling may be a feature specific to *P. o. wallikeri* but only in 30% of blood samples [9]. Clinical differences between both species have not been clearly established, but *P. o. wallikeri* may have a shorter period of latency [13].

Recently, a multicentre, retrospective study in Spain was reported, comparing 21 imported *P. o. curtisi* and 14 *P. o. wallikeri* infections confirmed by PCR and gene sequencing [5]. The only significant finding was more severe thrombocytopaenia among patients with *P. o. wallikeri* infection than among those with *P. o. curtisi* (p = 0.031). However, non-significant trends showing shorter latency, lower albumin level, higher temperature and markers of more severe haemolysis were found in *P. o. wallikeri* group. Because of the retrospective design and small number of patients, larger, prospective studies were warranted to confirm these findings. Therefore, the results of a multicentre, prospective, comparative study of imported *P. o. wallikeri* and *P. o. curtisi* infections conducted in several European countries during the period 2014–2017 are presented below.

## Methods

### Study design

A prospective, observational, un-randomized, open-label, multicentre study was performed at European hospitals of TropNet Europe, European Network for Tropical Medicine and Travel Health. Participants were recruited between March 2014 and May 2017. The study was approved by the University Hospital Príncipe de Asturias Ethical Board and at each site as needed. Written informed consent was obtained from the participant or a legal representative before enrolment in the study. The trial was conducted in accord with the Declaration of Helsinki and Good Clinical Practice.

### Microbiologic diagnosis

The initial diagnosis of imported *P. ovale* spp. infection was made up by thick and/or thin film and/or second generation RDT and/or PCR available in each hospital. Blood smears were stained by a standard technique with Giemsa solution for 30 min and were reviewed by an expert microbiologist. Parasite count was measured by determining the proportion of parasitized erythrocytes or the number of trophozoites per microlitre. Mixed infections with other *Plasmodium* species were excluded.

### Laboratory samples

For cases tested positive, three drops (≈ 50 μL) of full blood left after routine laboratory tests, were dotted on filter paper (Whatman™) and sent by regular mail to the Reference Malaria & Emerging Diseases Laboratory of the National Centre of Microbiology in Madrid. If DNA of *P. ovale* spp. had already been isolated, this was sent into a 1.5 mL screw cap tube with complete closure to the same Reference Laboratory.

### Isolation of parasite DNA and molecular diagnosis confirmation

DNA isolation was performed using the QIAamp DNA blood mini kit (QIAGEN^®^) from whole blood following the manufacturer’s protocol. Molecular diagnosis confirmation of *P. ovale* spp. was carried out by semi-nested multiplex malaria PCR [18] which allows to distinguish the four more prevalent human malaria species.

### *Plasmodium ovale* subspecies characterization and confirmation

Characterization of *P. o. curtisi* and *P. o. wallikeri* was performed by partial sequencing of the ssrRNA gene and ssrRNA amplification was performed by a nested PCR assay specific for *Plasmodium* genus. The first reaction included UNR (5′-GACGGTATCTGATCGTCTTC-3′) and PLF (5′-AGTGTGTATCCAATCGAGTTTC-3′) primers [18]. The second PCR reaction incorporates the products of the first reaction along with NewPLFsh (5′-CTATCAGCTTTTGATGTTAG-3′) and NewRevsh (5′-CCTTAACTTTCGTTCTTG-3′) primers. Infection with different malaria species yields products of different sizes between 710 and 740 bp.

The PCR mixture in both reactions consisted of 75 mM TrisHCl (pH 9.0), 2 mM MgCl_2_, 50 mM KCl, 20 mM (NH_4_)_2_SO_4_, 200 μM of dNTP, 0.075 µM of each primers, 1.25 units of *Taq* DNA polymerase (Biotools B&M Labs., S.A., Madrid, Spain), and 5 μL of DNA, extracted by QIAgen kit, was used as template in a reaction volume of 50 μL. For the second reaction mixture, 2 µL of the PCR product of the first reaction was used as template. For both reactions, a GeneAmp^®^ PCR System 2700 thermal cycler (Applied Biosystems Laboratory) was used, beginning with 7 min at 94 °C, followed by (first-round) 40 cycles of 20 s at 94 °C, 20 s at 62 °C, and 30 s at 72 °C, or (second-round) 35 cycles of 20 s at 94 °C, 20 s at 53 °C, and 20 s at 72 °C. The final cycle was followed by an extension time of 10 min at 72 °C.

The amplified products were purified using Illustra DNA and Gel Band Purification Kit (*General Electric Healthcare*) and sequenced with the Big Dye Terminator v3.1 Cycle Sequencing in an ABI PRISM^®^ 3700 DNA Analyzer. All amplified products were sequenced in both directions twice.

In order to confirm *P. ovale* subtyping, a nested PCR amplification plus sequencing targeting cytochrome b was performed in three samples of each group.

### Data collection

An anonymized database was designed to input all the medical information and laboratory registries in a prospective way. Data collected included gender, age, ethnicity, underlying diseases, type of patient, dates and purpose of travel, countries visited, malaria chemoprophylaxis, date of admission and diagnosis, presenting clinical signs or symptoms and complications of severe malaria according to criteria of the World Health Organization (WHO) [19] where no threshold of parasitaemia for *P. ovale* spp. was established. The closest possible date of inoculation was defined as the day of departure from a malaria endemic area. The time between date of arrival and onset of illness or diagnoses was calculated once asymptomatic patients were excluded. Patients were classified as an early immigrant if they had stayed in a country without malaria for less than a year prior to diagnosis. An immigrant was a VFR (visiting friends and relatives) if travelled to a country endemic of malaria after a year of stay in a non-malarial area, and VFR traveller if visited his/her first-degree relative’s country of birth having been born in a non-malaria endemic country. Recent *Plasmodium* infection was defined as probable or definite malaria infection in the previous 12 months before *P. ovale* spp. diagnosis.

Laboratory results included microbiological data with parasitaemia count, full blood count with white blood cells, haemoglobin and platelets, values of glucose, creatinine, albumin, transaminases, lactate dehydrogenase (LDH), total bilirubin (tBR) in plasma, the coagulation parameters activated partial thromboplastin time (APTT) and international normalized ratio (INR), glucose-6-phosphate dehydrogenase (G6PDH) activity in red blood cells, as well as serological studies of infection with human immunodeficiency virus (HIV), hepatitis B (HBV) and hepatitis C (HCV). Thrombocytopaenia was defined as a platelet count under 150,000/µL. Treatments, clinical and microbiological evolution and duration of hospital stay of those admitted were recorded. Treatments were performed following the guidelines of each country and health centre without a common recommendation.

### Statistical analysis

Differences of proportions were evaluated by the Chi-squared-test if less than 20% of cells had five or less expected values. If more than 20% of cells had five or less expected values we used the Fisher’s exact test for categorical variables. Differences of means between groups were calculated by the Student’s *t*-test for independent samples if the normal distribution could be assumed. In the Student’s t test for independent samples, the Levene’s test for homogeneity of variances was used. If normal distribution was not valid, the non-parametric Mann–Whitney *U*-test was performed. To test for normality, either the Shapiro–Wilks for small samples or the Kolmogorov–Smirnov with Lilliefors’ correction for large samples were used. Values are reported as number of patients and percentage or, for non-parametric distributions, as median and interquartile range (IQR). A multivariate linear regression analysis was performed for continuous variables and multivariate logistic regression for categorical variables to confirm if real differences were found when non-homogenous population between both groups were identified. A two-sided *p* value of 0.05 or less was considered to indicate statistical significance. Statistical analysis was performed using the SPSS version 21 (SPSS Inc, Chicago, IL, USA).

## Results

During the period of the study, a total of 96 blood samples from 29 hospitals were sent to the reference laboratory. Of them, 13 were excluded because of mixed infections (11 with *P. falciparum*, 1 with *P. vivax* and 1 with *Plasmodium malariae*), 2 samples did not get amplified and data from another 2 were not obtained. At the end, 79 correctly genotyped and with complete patient information were included for statistic analysis. Of them, 35 were identified as *P. o. curtisi* and 44 as *P. o. wallikeri.*

Table 1 presents demographic and epidemiological data from both groups. A significant difference in the distribution of gender, ethnicity and type of patients was shown. Males clearly predominate in wallikeri group whereas gender distribution was more evenly matched in the curtisi group. Conversely, on ethnicity, African patients were 74.3% of curtisi group. This ethnic distribution is reflected in the type of patient with a majority of VFR and recent immigrants in the *P. o. curtisi* infection versus a larger number of displaced for working or cooperation in the other species. The remaining epidemiological data did not differ including country of infection, duration of travel, chemoprophylaxis, latency time, time until diagnoses, recent *Plasmodium* infection or underlying diseases. There is a wide range of 21 countries as the place of infection, most of them in West Africa.

**Table 1:** Demographic and epidemiological characteristics

	*Plasmodium ovale curtisi* (n=35)	*Plasmodium ovale wallikeri* (n=44)	*p*
Sex			*0.028*
Male	17 (48.6)	32 (72.7)	
Female	18 (51.4)	12 (27.3)	
Age, median years (IQR)	35 (23.0–53.0)	38.5 (22.2–52.7)	0.529
Ethnicity			*0.017*
African	26 (74.3)	21 (47.7)	
Caucasian	9 (25.7)	23 (52.3)	
Type of patient			*0.034*
Early immigrant	11 (31.4)	4 (9.0)	
Visiting friends and relatives	14 (40.0)	17 (38.6)	
VFR traveller	1 (2.9)		
Tourism		3 (6.8)	
Work/cooperation	7 (20.0)	17 (38.6)	
Expatriate, long-term stay	2 (5.7)	3 (6.8)	
Country of infection			0.097
Equatorial Guinea	14 (40.0)	11 (25.0)	
Nigeria	5 (14.3)	6 (13.6)	
Cameroon	1 (2.9)	11 (25.0)	
Togo	1 (2.9)	1 (2.3)	
Liberia	2 (5.7)		
Tanzania		1 (2.3)	
Guinea-Conakry	1 (2.9)	1 (2.3)	
Ivory Coast	3 (8.6)	2 (4.5)	
Mozambique	1 (2.9)	2 (4.5)	
Congo	1 (2.9)	1 (2.3)	
Congo Democratic Republic		1 (2.3)	
Uganda	2 (5.7)		
Chad		2 (4.5)	
Ghana	1 (2.9)	1 (2.3)	
Sierra Leone		1 (2.3)	
Sudan	1 (2.9)		
Burundi	1 (2.9)		
Angola		1 (2.3)	
Mali		1 (2.3)	
Guinea-Bissau		1 (2.3)	
Madagascar	1 (2.9)		
Duration of travel, median days (IQR)	101 (21.0–240.0)	38.5 (26.7–157.5)	0.547
Chemoprophylaxis			0.078
No prophylaxis	14 (60.8)	24 (60.0)	
Incomplete	5 (21.7)	2 (5.0)	
Complete	4 (17.4)	14 (35.0)	
Time from arrival to onset of symptoms, median days (IQR)	34.0 (13.0–181.25)	30.5 (10.0–86.25)	0.171
Time from onset of symptoms to diagnoses, median days (IQR)	3 (0.0–10.0)	3.5 (0.7–8.0)	0.772
Recent *Plasmodium* infection	16 (51.6)	15 (40.5)	0.361
Other underlying conditions			1
G6PDH deficit	1/30 (3.3)	2/37 (5.4)	
Diabetes mellitus	1 (2.9)	4 (9.0)	0.3756
Drepanocytosis	1 (2.9)	1 (2.3)	
Pregnancy	1 (2.9)	0	
Chronic kidney disease	0	1 (2.3)	
Splenectomy	1 (2.9)	0	
Hepatitis B virus	0	3 (6.8)	
Hepatitis C virus	1 (2.9)	0	
HIV	3 (8.6)	1 (2.3)	

Values are no. (%) or no. positive/total no. (%) and median (interquartile range). Italicface indicates significance

*IQR* interquartile range, *VFR* visiting friends and relatives, *G6PDH* glucose-6-phosphate dehydrogenase, *HIV* human immunodeficiency virus

Microbiological and laboratory information is reported in Table 2. Several analytical parameters showed statistically significant differences such as total leukocyte and platelet count, number of thrombocytopaenic patients, haemoglobin, creatinine, LDH or INR values. Even after the multivariate study was performed, eliminating the confounding factor of ethnicity, the number of platelets, thrombocytopaenic patients and INR remained significant (Table 3). Latency time was also included in the multivariate analysis because of the significant difference found in a previous report [13]. A statistically shorter latency period also appeared, but only in Caucasians patients with *P. o. wallikeri* compared to those in the curtisi group. On the other hand, no difference in parasitological data including submicroscopic infection, parasitaemia or sensitivity of the common antigen of RDT was seen.

**Table 2:** Microbiological and analytical characteristics

	*P ovale curtisi* (n = 35)	*P ovale wallikeri* (n = 44)	*p*
Positive thick smear	28/34 (82.4)	36/42 (85.7)	0.689
Only PCR positive	5 (18.5)	6 (19.4)	1
Parasitaemia, median × µL (IQR)	2550 (647.5–11,677.5)	946 (600–4450)	0.441
Parasitaemia index			0.275
Very low ≤ 0.01%	3/22 (13.6)	7/28 (25.0)	
Low 0.02–0.1%	8/22 (36.4)	13/28 (46.4)	
Medium > 0.1%	11/22 (50.0)	8/28 (28.6)	
Rapid diagnostic test			
Common antigen positive	6/23 (26.1)	14/33 (42.4)	0.209
*P. falciparum* antigen positive	0/23 (0.0)	3/33 (9.1)	0.502
Total WBC count, median cells/µL (IQR)	5600 (4600–6270)	4460 (3827–6015)	*0.049*
Haemoglobin level, median g/dL (IQR)	12.1 (10.1–13.5)	13.2 (11.5–14.1)	*0.032*
Platelet count, median cells/µL (IQR)	130,000 (81,000–281,000)	105,500 (69,000–141,500)	*0.039*
Platelet count < 150,000 cells/µL	19 (54.3)	34 (79.1)	*0.016*
Albumin level, median g/dL (IQR)	3.60 (3.20–4.00)	3.38 (3.02–3.73)	0.139
Creatinine level, median mg/dL (IQR)	0.79 (0.59–1.00)	0.95 (0.75–1.10)	*0.030*
LDH level, median value × IU/L (IQR)	267.0 (221.2–367.2)	370.0 (256.0–508.7)	*0.028*
AST level, median IU/L (IQR)	27.00 (20.00–32.50)	25.50 (19.90–43.75)	0.691
ALT level, median IU/L (IQR)	30.00 (18.00–43.20)	24.00 (18.00–48.00)	0.648
Total bilirubin level, median mg/dL (IQR)	0.83 (0.60–1.35)	1.22 (0.70–1.80)	0.086
INR, median (IQR)	1.08 (0.96–1.17)	1.12 (1.10–1.20)	*0.019*
APTT, median (IQR)	29.50 (25.10–37.07)	30.65 (26.27–32.97)	0.762
Glucose, median mg/dL (IQR)	91.5 (83.0–103.7)	97.0 (82.0–113.0)	0.468

Values are no. (%) or no. positive/total no. (%) and median (interquartile range). Italicface indicates significance

*IQR* interquartile range, *PCR* polymerase chain reaction, *WBC* white blood cells, *LDH* lactate dehydrogenase, *AST* aspartate aminotransferase, *ALT* alanine aminotransferase, *INR* international normalized ratio, *APTT* activated partial thromboplastin time

**Table 3:** Multivariate linear regression and multivariate logistic regression analysis related to ethnicity of parameters with significant differences in univariate analysis and latency time

	Regression coefficients	95% confidence interval	*p*
Total WBC count, ×10^9^ cells/L	− 850.884	− 1931.456 to 229.689	0.121
Haemoglobin level, g/dL	− 0.903	− 0.085 to 1.891	0.073
Platelet count cells/µL	− 39,707.641	− 71,351.311 to − 8063.972	*0.015*
Creatinine level, mg/dL	0.095	− 0.024 to 0.215	0.117
LDH level, IU/L	12.537	− 160.156 to 185.230	0.885
INR	0.077	0.003–0.151	*0.041*
Time from arrival to onset of symptoms, days			
African patients	− 41.108	− 127.135 to 44.920	0.344
Caucasian patients	− 75.001	− 144.040 to − 5.962	*0.034*

	Odds ratio	95% confidence interval	*p*
Platelet count < 150,000 cells/µL	0.314	0.117–0.847	*0.022*

Values show the relationship of *P. o. wallikeri* respect to *P. o. curtisi.* Italicface indicates significance

*WBC* white blood cells, *LDH* lactate dehydrogenase, *INR* international normalized ratio

In respect of the clinical manifestations and time of hospitalization no differences were found in (Table 4). Treatments were widely different according to the protocols of each centre. Primaquine was supplied to most patients in both groups and only three patients showed G6PDH deficiency that contraindicated this treatment. Five patients showed analytical criteria for severe malaria and another one a rare and severe complication (haemophagocytosis). Outcome in nearly all cases was good, although two patients did not receive any anti-malarial treatment due to loss of follow-up.

**Table 4:** Clinical and therapeutic characteristics

	*P ovale curtisi* (n=35)	*P ovale wallikeri* (n=44)	*p*
Asymptomatic	2 (5.7)	2 (4.5)	1
Fever	32 (91.4)	39 (88.6)	1
Headache	17 (48.6)	29 (65.9)	0.121
Nausea	9 (25.7)	11 (25.0)	0.942
Arthralgia	10 (28.6)	15 (34.1)	0.600
Myalgia	17 (48.6)	18 (40.9)	0.496
Vomitus	4 (11.4)	12 (26.3)	0.082
Abdominal pain	5 (14.3)	3 (6.8)	0.460
Diarrhea	4 (11.4)	5 (11.4)	1
Other (cough, dyspnea, chest pain, dizziness)	9 (25.7)	10 (22.7)	0.758
Duration of hospitalization, median days (IQR)	4 (2.0–6.0)	3 (3.0–6.0)	0.713
Treatment			0.826
Chloroquine	17 (48.6)	18 (40.9)	
Atovaquone/proguanil	12 (34.2)	11 (25.0)	
Quinine + doxycycline		2 (4.5)	
Atovaquone/proguanil + chloroquine	1 (2.9)	1 (2.3)	
Artesunate + artemether–lumefantrine	1 (2.9)		
Dihidroartemisinin–piperaquine	3 (8.6)	7 (15.9)	
Artemether–lumefantrine		1 (2.3)	
Artesunate + chloroquine		1 (2.3)	
Artesunate + atovaquone/proguanil	1 (2.9)		
Artesunate		1 (2.3)	
Lost to follow up, no treatment		2 (4.5)	
Primaquine	25 (71.4)	34 (81.0)	0.325
Severe malaria and complications			1
Total bilirubin > 3 mg/dL and haemoglobin < 7 g/dL	1 (2.9)^a^		
Total bilirubin > 3 mg/dL		3 (6.8)^b^	
Haemoglobin < 7 g/dL	1 (2.9)		
Haemophagocytosis		1 (2.3)	
Exitus	0	0	1

Values are no. (%) or no. positive/total no. (%) and median (interquartile range)

*IQR* interquartile range

^a^This patient had drepanocytosis

^b^One patient had drepanocytosis and G6PDH deficit and other patient had diabetes

## Discussion

This is the largest prospective study to date on the two species of infection by imported *P. ovale* trying to overcome recruitment difficulties and limitations of previous retrospective studies.

On the results obtained, significant differences were found in the epidemiological characteristics of sex, ethnicity and type of patients between both groups. Ethnic differences may be the main confounding factor since there is a greater probability that African patients retain some semi-immunity against *P. ovale* spp. infection (excluding VFR travellers), which would generally result in less clinical and analytical involvement [20]. On the other hand, the difference in sex distribution and type of patients seems to be clearly related to the ethnic origin since in a subgroup analysis, 16 out of 18 (89%) of the patients with *P. o. wallikeri* who travelled by international cooperation were male and Caucasians, which would bias the characteristics of this group.

This distribution could be a result of chance alone or due to the fact that actually Caucasians patients are more susceptible to infection by wallikeri parasites. However, a previous retrospective study did not show these differences in sex, ethnicity or type of patient [5] and the few studies of imported ovale malaria reporting a significant number of patients showed either equality of sexes (but did not report on ethnicity) [13] or they were nearly all Chinese men who worked in African countries [10].

When performing the multivariate study adjusted by ethnicity to reduce this confounding factor, the number of patients with thrombocytopaenia and two of the analytical alterations remained significant. Thrombocytopaenia was more pronounced in *P. o. wallikeri* and INR values were higher in the wallikeri group. The comparation of the rest of analysis variables were not finally significant, over all those that indicate a greater degree of haemolysis, such as LDH, tBR or haemoglobin level.

In the case of thrombocytopaenia, these results confirm the main finding of a previous retrospective study where the only difference was more severe thrombocytopaenia in *P. o. wallikeri* infection [5]. Thrombocytopaenia is a common finding in patients with malaria of all *Plasmodium* species ranging from 24 to 94% of indicence, although spontaneous haemorrhages are infrequent and limited to very severe cases [21]. Also it is of note that thrombocytopaenia seems to be a very common finding in *P. ovale* spp. infection. Of the 79 patients analysed, 53 (67%) had under 150,000 platelets/µL, which corresponds to our previous series findings of 66.6% in 16 imported ovale infection patients [22] and a retrospective study where 26 out of 35 patients (75.3%) suffered from thrombocytopaenia [5]. Only one patient in each group showed under 50,000 platelets/µL again without any haemorrhagic feature.

The pathogenesis of thrombocytopaenia in malaria is not fully understood but is accepted that encompasses several different mechanisms that lead to increased platelet destruction or consumption, such as increased attachment to endothelium and adherence to Von Willebrand factor, clumping and agglutination of infected and uninfected erythrocytes, consumption into the coagulation process and haemolysis, increased diffuse platelet sequestration, decreased nitric oxide bioavailability, and immune complexes-mediated destruction [23].

Although thrombocytopaenia is not among the severity criteria of the WHO, evidence is accumulating that it can predict an adverse outcome, which seems to be driven by a greater severity of illness in *P. falciparum* or *P. vivax* [21, 24] and severe thrombocytopaenia can identify an increased risk of death from falciparum or vivax malaria [25]. Therefore, in the case of *P. o. wallikeri* it could indicate a greater intrinsic pathogenicity of this species compared to *P. o*. *curtisi,* although without evidence of any major significantly worse clinical criteria of severity.

Regarding the other significant finding of this study, the higher INR elevation in *P. o. wallikeri* compared to *P. o. curtisi*, this increase was always mild and in no case exceeded the value of 1.54 nor was related to any bleeding. On the other hand, the APTT values were similar in both groups. No other coagulation factors, fibrinogen levels, fibrin degradation products or d-dimer were measured that could argue in favour of a significant coagulation disorder.

It is well known that *P. falciparum* malaria is associated with significant coagulation activation. The increase in INR or its equivalent, prolongation of prothrombin time, is produced by the activation of the extrinsic pathway of the coagulation cascade and can lead to disseminated intravascular coagulation in the most severe cases of malaria. *Plasmodium falciparum* seems to trigger coagulation activation through multiple different pathways although the mechanisms involved are not well understood [26]. Much less information on coagulation is available in *P. ovale* spp. infection. In the case of INR, the finding of this study might support the hypothesis of a more pathogenic wallikeri parasite, but does not seem to pose a significant clinical difference or severity.

In the initial analysis, no differences were found in the latency period, but when the multivariate analysis was performed a significant shorter time appeared in *P. o. wallikeri* compared to *P. o. curtisi* infection, but only in Caucasians patients. This difference is more significant since only one of the seven Caucasians patients who suffered infection by *P. o. curtisi* had taken adequate prophylaxis versus 11 out of 22 (50%) of the wallikeri group, which should have lengthened their latency. The retrospective study by Nolder et al. in the UK also found significant shorter latency period in wallikeri cases but did not report the ethnicity or patient characteristics [13]. On the contrary, another study in Chinese people (ethnically homogeneous but with unknown previous exposure to malaria or chemoprophylaxis use) encompassing 109 *P. o. curtisi* and *P. o. wallikeri* [10] imported from Africa, did not find any difference in latency. In a previous retrospective study, only a trend to shorter wallikeri latency was found (p = 0.07) [5].

These results of different latency times in only Caucasians patients suggest a strong influence of the previous partial immunity to malaria in the time of symptoms of *P. ovale* spp. infection. Finally, note that in both species latencies greater than 1 year have been found as described previously [11].

A complete chemoprophylaxis was reported in 22.8% of ovale spp. infections and up to 35% in *P. o. wallikeri*, a high percentage if compared with data from large series of imported malaria as in USA 2014, where only 7.8% of travellers correctly took prophylaxis [11]. If analysed by *Plasmodium* species, these results are similar to the study by Nolder et al. which stated that the proportion of ovale malaria which occurred in patients reporting chemoprophylaxis use (33%) was significantly higher than for *P. falciparum* (6.4%) and *P. vivax* (23.7%) [13]. Also, in a USA and Israel study, up to 73% of imported *P. ovale* spp. used an effective prophylaxis [27]. Moreover, in a recent Spanish study considering only the subgroup of travellers with *P. ovale*/*P. vivax* malaria, these patients had also taken chemoprophylaxis significantly more frequently than those with non-*P. ovale*/*P. vivax* malaria (35.5% vs. 2.7%) [28]. These results reinforce the idea that current prophylaxis does not adequately act on *P. ovale* spp. infection, since it is ineffective against its hypnozoites and, therefore, does not prevent a delayed primary attack or true relapses from occurring when there is no prophylactic medication in blood.

Although the information cannot be completely reliable, over 40% of patients reported an episode of malaria in the previous 12 months. This could have been a primary infection by *P. ovale*, another ovale relapse, a mixed infection with other *Plasmodium* or simply a non-ovale malaria unrelated to the current episode. Since it is not feasible to obtain a previous diagnostic sample, it is impossible to know if the current episode represented a primary infection or a relapse by *P. ovale*. Primary infection could only be diagnosed in the cases of first malarial episode and very short latency period.

Regarding the underlaying conditions, it is worth focusing on diabetes mellitus and drepanocytosis. The prevalence of diabetes mellitus is increasing worldwide, especially in low income countries, as are found in sub-Saharan Africa [29]. The number of diabetics in this study is as high as 6.32% (5/79) in a population with a low average age, especially in wallikeri group. In a retrospective curtisi–wallikeri study up to 8.5% of patients suffered from diabetes [5] and in a previous study of imported *P. ovale* infection 3 out of 16 patients (18.7% with mean age 30.6 years) were also diabetics [22]. A recent Swedish study, reported diabetes in 3.5% of 937 patients with imported *P. falciparum* and 2.5% in 398 imported malaria in a Spanish Tropical Medicine Centre [30]. In a series of 229 *P. falciparum* infections from 2006 to 2015 in Hospital Universitario Príncipe de Asturias (HUPA), 2.6% were diabetics (unpublished data). As diabetes is a known risk factor for malaria due to *P. falciparum* in endemic areas [31], the higher prevalence of this chronic disease in *P. ovale* studies might indicate that would also be a risk factor, and deserves further investigation to confirm this finding.

In endemic areas, carrying the sickle cell trait represents a risk factor for infection by *P. ovale* spp. [20] and a partial protective factor against severe *P. falciparum* infection. In this study, 2 out of 47 African patients (4.2%) had drepanocytosis. Moreover, in the previous comparative retrospective study, 8.3% (2 out of 24) showed drepanocytosis and up to 23% (3/13) in the series of 16 imported *P. ovale* spp. [22]. Gathering African patients from the three studies (7/84) and comparing to 1.35% of patients in 221 African people with imported *P. falciparum* in HUPA during the period 2006–2015 (unpublished data) significant difference can be found (p < 0.005) that would support the results of the study conducted in endemic area.

As in previous studies, no differences were detected in parasitaemias between both groups [6, 9, 32]. Most of them had low parasite counts clearly below 1%, making it even more difficult to find statistically real differences. Surprisingly, two patients reached 2% parasitaemia, a Caucasian traveller without any immunity to malaria and a child with drepanocytosis and intense anaemia that enhanced the proportion of infected red blood cells. Such high parasitaemia has been rarely described, sometimes linked to serious complications such as spleen rupture [33]. Finally, the absence of Schüffner’s stippling in blood smear, which has been described as a possible feature specific to *P. o. wallikeri*, was not studied.

There was also no difference in the sensitivity of RDTs, which was low as described in the literature [2] without exceeding 45% in either group. Almost 20% of the cases in both groups were diagnosed only by PCR, as RDT and/or thick blood smears were negative. In endemic areas this percentage is much higher, especially in asymptomatic patients [3]. Therefore, if the clinical and epidemiological suspicion is high, it would be convenient to repeat the diagnostic tests and request molecular PCR techniques not to miss *P. ovale* spp. diagnosis.

This study confirms that this infection can cause malaria with severity criteria or complications, in this case 7.6% of all patients with no differences between both species. In series of imported malaria by *P. falciparum* the rate of severe cases was around 2–17% [11, 34], but with a much higher incidence of life-threatening complications, such as ADRS, severe anaemia or cerebral malaria than described in *P. ovale* spp. infection [8]. A rare case of haemophagocytosis was included that had been described only in *P. falciparum* and *P. vivax* infection [35], but not in *P. ovale* spp. Analysing the six patients with severe malaria or complications, two had drepanocytosis (which also produces anaemia) and one was diabetic, which might indicate the possible importance of these risk factors in the severity of *P. ovale* spp. infection.

In cases of malaria with tBR > 3 mg/dL, the WHO severity criteria add parasitaemia levels above 100,000/µL in *P. falciparum* and > 20,000/µL in *P. vivax* and *Plasmodium knowlesi,* but is not known in the case of *P. ovale* spp. In this study, all four cases with tBR > 3 mg/dL had parasitaemias over 4000/µL. In addition, four patients received parenteral artesunate, two of them with tBR > 3 mg/dL and one with unusually high parasitaemias of 2%. It is described that host response may reach full strength at lower parasitaemia in *P. vivax* and *P. ovale* spp. infection than in *P. falciparum* [36] so, perhaps it would be also convenient to establish a threshold of parasitaemia with jaundice and hyperparasitaemia in *P. ovale* spp. in the face of treatment decisions and outcome.

The treatments were very varied, including artemisinins alone or in combination, atovaquone/proguanil, chloroquine or quinine-doxycycline, all with good initial clinical evolution, which is compatible with the sensitivity of *P. ovale* spp. described for multiple antimalarials [8]. Over 70% of them received primaquine with good tolerance, although there was no long-term follow-up recorded to assess episodes of relapse or late recrudescence.

The prospective study design has minimized some of the limitations of the previous retrospective study but some of them still remain. First, although it is one of the largest studies carried out worldwide, the number of patients may still lack sufficient statistical power to show other differences between infections with both *P. ovale* species. Second, only strains of *P. ovale* from Africa, and patients from Africa and Europe were analysed; a study of infections and patients from Asia, Oceania or other places might show different results. Third, the behaviour of these species in endemic countries may be different but it would be difficult to gather a significant number of patients with *P. ovale* spp. monoinfection due to the high prevalence of mixed *Plasmodium* infections and malaria reinfections. Fourth, the date of infection is a minimal approximate and it is virtually impossible to distinguish a primary infection from a relapse. Last, a mix of ethnicities, non-immune and semi-immune patients led to heterogeneous study groups, although the multivariate statistical study has reduced this bias.

## Conclusions

In this study, imported *P. o. wallikeri* infection was more frequent in males and Caucasian patients than in *P. o. curtisi* group. Malaria caused by *P. o. wallikeri* produced more thrombocytopenic patients, more marked thrombocytopaenia and a higher INR than *P. ovale curtisi*, although without other clinical or severity differences. *Plasmodium o. wallikeri* malaria had shorter latency, but only in Caucasian patients. These findings suggest that wallikeri species might be somewhat more pathogenic than *P. ovale curtisi*. Some cases with severity criteria were seen in both species and required treatment with intravenous artesunate. The clinical evolution with any anti-malarial drug was usually good, however, chemoprophylaxis seemed less effective in *P. ovale* spp. infection than in the case of *P. falciparum*. Diabetes mellitus might also be a risk factor for *P. ovale* spp. infection such as drepanocytosis. Current RDT was shown to have low diagnostic sensitivity in both species and PCR was essential for diagnosis in a significant percentage of cases.

## Abbreviations

ADRS: acute respiratory distress syndrome
APTT: activated partial thromboplastin time
DNA: deoxyribonucleic acid
G6PDH: glucose-6-phosphate dehydrogenase
HBV: hepatitis B virus
HCV: hepatitis C virus
HIV: with human immunodeficiency virus
INR: international normalized ratio
IQR: interquartile range
LDH: lactate dehydrogenase
PCR: polymerase chain reaction
RDT: rapid diagnostic test
ssrRNA: small-subunit ribosomal ribonucleic acid
tBR: total bilirubin
VFR: visiting friends and relatives
WHO: World Health Organization
